# S07-4 Sporting programs aimed at inactive population groups in the Netherlands: factors influencing their long-term sustainability in the organized sports setting

**DOI:** 10.1093/eurpub/ckac093.037

**Published:** 2022-08-29

**Authors:** Linda Ooms

**Affiliations:** Mulier Instituut, Utrecht, The Netherlands

**Keywords:** Sports club, national sports federation, sporting program, inactive, sustainability

## Abstract

**Background:**

The organized sports sector has received increased interest as a setting to stimulate physical activity among inactive target groups. To include many inactive people and to obtain population health benefits, it is important that effective sporting programs are sustained (i.e. continuation of program activities) over a long period of time. This study identified the factors influencing the long-term sustainability of these kind of sporting programs located within local sports clubs in the Netherlands.

**Methods:**

Fourteen Dutch sporting programs aimed at increasing physical activity levels of inactive population groups and funded within the National Action Plan for Sport and Exercise (NAPSE) were the focus of this study. The programs were developed by ten Dutch National Sports Federations (NSFs) and implemented by different sports clubs in the Netherlands within a three-year funded implementation period (2008-2011). This research consisted of semi-structured face-to-face interviews with the program coordinators of the NSFs (n = 14) and semi-structured telephone interviews with representatives of sports clubs that provided the programs (n = 17 continued the program, n = 11 discontinued the program) six and a half years after the funding period ended (November 2017-March 2018). A sustainability framework with five pre-specified main themes (i.e. program design, implementation, trainer/coach, organizational setting, broader community environment) guided data collection and (deductive) thematic analysis.

**Results:**

Ten of the fourteen NAPSE funded sporting programs were sustained at the level of the NSFs. Most factors facilitating (+) and impeding (-) the long-term sustainability of the programs were common to both NSFs and sports clubs, like program adaptation (+) and a lack of program financing (-). Program evaluation (+) and high program costs (-) were specific factors mentioned by NSFs, while factors related to human resources (e.g. lack of volunteers (-)) or the sports club nature (e.g. social aspect in program design (+)) applied to sports clubs. The factors were summarized in the form of a checklist.

**Conclusions:**

Key factors influencing the long-term sustainability of the sporting programs were identified. The results can be used to develop strategies to promote long-term sustainability of these kind of programs and inform funding guidelines in countries with a similar organized sports infrastructure.

